# A clinical prognostic model for patients with esophageal squamous cell carcinoma based on circulating tumor DNA mutation features

**DOI:** 10.3389/fonc.2022.1025284

**Published:** 2023-01-05

**Authors:** Tao Liu, Mengxing Li, Wen Cheng, Qianqian Yao, Yibo Xue, Xiaowei Wang, Hai Jin

**Affiliations:** ^1^ Department of Thoracic Surgery, Changhai Hospital, Second Military Medical University, Shanghai, China; ^2^ Department of Medical Science, Shanghai AccuraGen Biotechnology Co., Ltd., Shanghai, China

**Keywords:** circulating tumor DNA, prognosis, nomogram, TNM staging system, esophageal squamous cell carcinoma

## Abstract

**Background:**

Few predictive models have included circulating tumor DNA (ctDNA) indicators to predict prognosis of esophageal squamous cell carcinoma (ESCC) patients. Here, we aimed to explore whether ctDNA can be used as a predictive biomarker in nomogram models to predict the prognosis of patients with ESCC.

**Methods:**

We included 57 patients who underwent surgery and completed a 5-year follow-up. With next-generation sequencing, a 61-gene panel was used to evaluate plasma cell-free DNA and white blood cell genomic DNA from patients with ESCC. We analyzed the relationship between the mutation features of ctDNA and the prognosis of patients with ESCC, identified candidate risk predictors by Cox analysis, and developed nomogram models to predict the 2- and 5-year disease-free survival (DFS) and overall survival (OS). The area under the curve of the receiver operating characteristic (ROC) curve, concordance index (C-index), calibration plot, and integrated discrimination improvement (IDI) were used to evaluate the performance of the nomogram model. The model was compared with the traditional tumor-nodes-metastasis (TNM) staging system.

**Results:**

The ROC curve showed that the average mutant allele frequency (MAF) of ctDNA variants and the number of ctDNA variants were potential biomarkers for predicting the prognosis of patients with ESCC. The predictors included in the models were common candidate predictors of ESCC, such as lymph node stage, angiolymphatic invasion, drinking history, and ctDNA characteristics. The calibration curve demonstrated consistency between the observed and predicted results. Moreover, our nomogram models showed clear prognostic superiority over the traditional TNM staging system (based on C-index, 2-year DFS: 0.82 vs. 0.64; 5-year DFS: 0.78 vs. 0.65; 2-year OS: 0.80 vs. 0.66; 5-year OS: 0.77 vs. 0.66; based on IDI, 2-year DFS: 0.33, *p <*0.001; 5-year DFS: 0.18, *p* = 0.04; 2-year OS: 0.28, *p <*0.001; 5-year OS: 0.15, *p* = 0.04). The comprehensive scores of the nomogram models could be used to stratify patients with ESCC.

**Conclusions:**

The novel nomogram incorporating ctDNA features may help predict the prognosis of patients with resectable ESCC. This model can potentially be used to guide the postoperative management of ESCC patients in the future, such as adjuvant therapy and follow-up.

## 1 Introduction

Esophageal cancer (EC) is a common malignant tumor of the digestive tract, with 604,100 new cases and 544,076 new deaths worldwide in 2020 (1). China accounts for approximately 50% of the global incidence, and esophageal squamous cell carcinoma (ESCC) accounts for >90% of all EC cases (1, 2). With the development of surgery, chemotherapy, radiotherapy, targeted therapy, and immunotherapy, the prognosis of EC has improved; however, it remains unsatisfactory (3, 4). Even early-stage patients who have undergone surgery relapse after a period of time. More than 80% of patients die of metastasis and recurrence, and the overall 5-year survival rate of EC is 15–25% (5, 6).

The tumor-nodes-metastasis (TNM) staging system has been used for decades to guide clinical treatment and predict the prognosis of patients with EC. The TNM staging system of EC is based on the pathological grade, depth of tumor invasion (pT), involvement of regional lymph nodes (pN), and distant metastasis (pM). However, owing to the heterogeneity of tumors, including ESCC, the survival of patients in the same tumor stage often varies in clinical practice (7). This fact indicates that other factors, including genomic features, may affect prognosis (8). TNM staging can predict treatment effects in some cases, but it is not catered to individualized patient evaluation (9). Thus, predicting survival using the TNM staging system is not ideal. Accordingly, predicting the prognosis of ESCC remains a challenging task, and identifying effective prognostic biomarkers or models is imperative to optimize the treatment and prognosis of patients with ESCC (10).

A nomogram is a predictive model developed using statistical methods that utilize current, real-world patient data. The predictive model can be updated as the patient is examined and tested, potentially leading to precision medical treatment (11). Nomograms integrate diverse prognostic and detective factors that can be used to determine individualized numerical probabilities of clinical outcomes (12). Currently, noninvasive detection and monitoring of diseases using circulating tumor DNA (ctDNA) is an active area of cancer research with considerable implications in clinical management (13). ctDNA, which is released from tumor cells into the blood circulation, reflects the genetic and epigenetic alterations of the original tumor and is an indicator of tumor burden and disease progression (14). Although some prediction models have been developed for patients with EC, these existing models mainly combine demographic and clinicopathological characteristics and have shown only moderate discriminative ability (11, 15, 16). We hypothesized that including ctDNA features may improve the performance of the models in predicting survival following surgery for EC.

In this study, we focused on exploring the association between the features of ctDNA mutation and the prognosis of patients with ESCC. We developed predictive models of disease-free survival (DFS) and all-cause overall survival (OS) based on ctDNA markers and clinicopathological factors that have been identified as independent prognostic predictors of resectable ESCC.

## 2 Material and methods

### 2.1 Study design

This study was conducted in Changhai Hospital, and we prospectively enrolled 75 patients with ESCC who underwent thoracic surgery from August 2015 to December 2016. The inclusion and exclusion criteria are listed in **Supplementary Table S1**. According to these criteria, 18 individuals were excluded, and 57 participants were finally included in the data analysis. This study was conducted in accordance with the Declaration of Helsinki and approved by the Institutional Review Board of Changhai Hospital. All participants provided written informed consent.

### 2.2 Sample collection and DNA isolation

Using K_2_-EDTA anticoagulant tubes (BD, Franklin Lakes, NJ, USA), we collected at least 10 mL of venous blood from each enrolled participant prior to surgery. Plasma samples were enriched using the double-spin protocol at 4°C (first: 1,900 × *g* for 10 min, upper phase; second: 16,000 × *g* for 10 min) and stored at -80°C until cell-free DNA (cfDNA) extraction. White blood cell (WBC) samples were separated from the blood after the first centrifugation (middle phase) and stored at -20°C until genomic DNA (gDNA) extraction. Tumor tissue samples were surgically collected, and 10 slides of formalin-fixed, paraffin-embedded (FFPE) sections were prepared for each case. We extracted cfDNA from plasma samples, gDNA from WBCs, and DNA from tumor tissues (tDNA) according to our previously reported methods (17). All isolated DNAs were then qualified and stored at -80°C until use.

### 2.3 Next-generation sequencing

For the capture-based, targeted next-generation sequencing (NGS), Accu-Act panel (61-gene; AccuraGen Inc., Shanghai, China) was used to sequence plasma cfDNA, tDNA, and gDNA. All sequencings were carried out by the Shanghai Yunsheng Medical Laboratory Co., Ltd. The sequencing, as well as the recall of mutations, was performed according to our previously reported methods (17, 18).

### 2.4 Statistical analysis and graphing

DFS and OS were measured from the date of the surgery to the date of cancer recurrence and any cause of death or the last follow-up (censored cases), respectively. The Kaplan–Meier method was used to estimate the median DFS and OS, and the log-rank test was used to analyze the survival curves of different groups. Cox univariate and multivariate analyses were performed to determine the independent prognostic factors, which were used to create survival hazard ratios. The two-sided statistical significance level was set at *p*<0.05. The nomograms were constructed using the results of the multivariate analysis. The area under the curve (AUC) and concordance index (C-index) were used to assess the discrimination ability of the nomogram. The marginal estimation and average prediction probability of the model were used to create the calibration curves. Furthermore, the nomograms were compared with the 8th edition of the American Joint Committee on Cancer (AJCC) TNM staging system in terms of AUC and integrated discrimination improvement (IDI). Statistical analysis and graphing were achieved by R version 4.1.1 (The R Foundation for Statistical Computing, Vienna, Austria) packages “ggplot2,” “survival,” “survminer,” “rms,” “timeROC,” “pROC,” “regplot,” and “survIDINRI.”

## 3 Results

### 3.1 Characteristics of the study cohort

**Figure 1:**
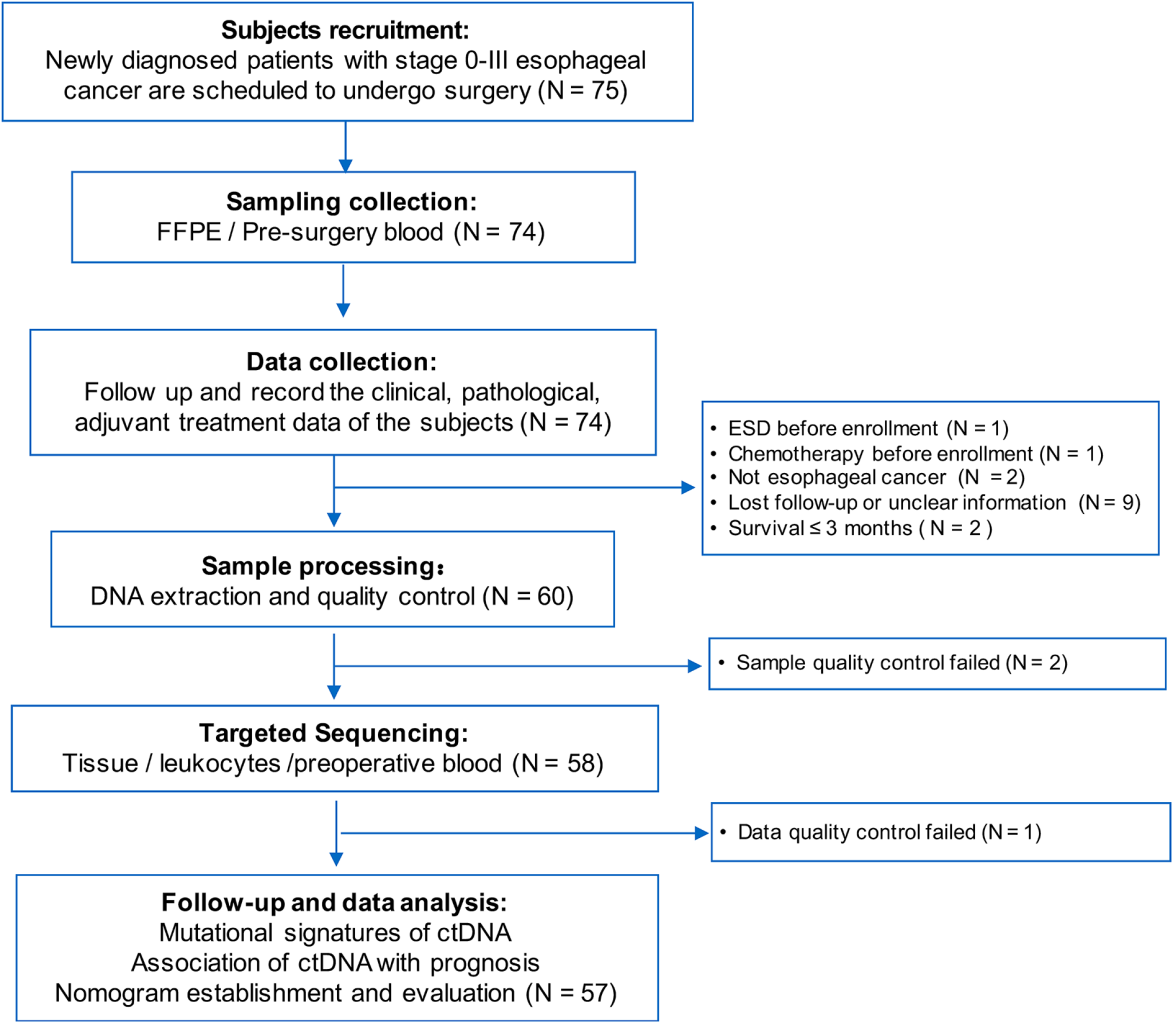
Patient selection flow of the esophageal squamous cell carcinoma cohort.

The study flowchart is shown in **Figure 1**. We initially enrolled 75 patients in this study. We excluded 18 patients who received neoadjuvant chemotherapy (n = 1), underwent primary endoscopic submucosal dissection prior to the first blood draw (n = 1), did not have ESCC (n = 2), survived for <3 months (n = 2), failed the sample quality control (QC) (n = 2) or sequencing data QC (n = 1), or were lost to follow-up (n = 9). In total, 57 participants were finally included in the data analysis (**Supplementary Table S2**). The clinicopathological characteristics and cfDNA features of the 57 patients are summarized according to recurrence status in **Table 1**. The study included 47 males (82.5%) and 10 females (17.5%), and the mean age of the patients was 64.8 ± 7.4 years. Additionally, 54.4% and 52.2% of the patients had a history of smoking and drinking, respectively.

**Table 1:** Clinical and cfDNA characteristics of participants.

Variables	Whole cohort		Two-year recurrence status			Five-year recurrence status		
	(n = 57)		No (n = 36)	Yes (n = 21)	P value	No (n = 21)	Yes (n = 36)	P value
Demographic information								
Age					0.76			0.72
Mean ± SD* (year)	64.8 ± 7.4		64.6 ± 6.9	65.2 ± 8.2		64.3 ± 6.1	65.1 ± 8	
Sex					0.30			1.00
Female	10 (17.5)		8 (22.2)	2 (9.5)		4 (19)	6 (16.7)	
Male	47 (82.5)		28 (77.8)	19 (90.5)		17 (81)	30 (83.3)	
Body mass index					0.30			0.15
Mean ± SD	23.14 ± 2.62		23.4 ± 2.6	22.7 ± 2.7		23.8 ± 2.6	22.8 ± 2.6	
Smoking history					0.16			0.82
No	26 (45.6)		19 (52.8)	7 (33.3)		10 (47.6)	16 (44.4)	
Yes	31 (54.4)		17 (47.2)	14 (66.7)		11 (52.4)	20 (55.6)	
Drinking history					**0.06**			**0.05**
No	31 (54.4)		23 (63.9)	8 (38.1)		15 (71.4)	16 (44.4)	
Yes	26 (45.6)		13 (36.1)	13 (61.9)		6 (28.6)	20 (55.6)	
Clinical and pathological information								
Tumor location					**0.05**			**0.02**
Lower	13 (22.8)		5 (13.9)	8 (38.1)		1 (4.8)	12 (33.3)	
Upper-Middle	44 (77.2)		31 (86.1)	13 (61.9)		20 (95.2)	24 (66.7)	
Surgical procedure					0.19			**0.03**
Ivor–Lewis	43 (75.5)		27 (75)	16 (76.2)		14 (66.7)	29 (80.6)	
Mckeown	10 (17.5)		8 (22.2)	2 (9.5)		7 (33.3)	3 (8.3)	
Sweet	4 (7.0)		1 (2.8)	3 (14.3)		0 (0)	4 (11.1)	
Maximum tumor diameter					**0.01**			**0.03**
Mean ± SD (cm)	3.62 ± 1.56		3.3 ± 1.4	4.3 ± 1.7		3 ± 1.4	4 ± 1.6	
Tumor volume					**0.05**			0.16
Median (IQR*) (cm^3^)	9 (4.8–17.5)		8.3 (4.1–13.9)	12 (7.5–20)		9 (3.5–13.5)	9.4 (6,20.1)	
Tumor differentiation**^#^**					0.14			0.18
Tis-G1	11 (19.3)		9 (25.7)	2 (9.5)		6 (30)	5 (13.9)	
G2	37 (64.9)		23 (65.7)	14 (66.7)		13 (65)	24 (66.7)	
G3	8 (14.0)		3 (8.6)	5 (23.8)		1 (5)	7 (19.4)	
Depth of invasion					0.23			**0.004**
pTis-pT1	14 (24.6)		11 (30.6)	3 (14.3)		9 (42.9)	5 (13.9)	
pT2	23 (40.3)		15 (41.7)	8 (38.1)		10 (47.6)	13 (36.1)	
pT3	20 (35.1)		10 (27.8)	10 (47.6)		2 (9.5)	18 (50)	
Lymph node stage					0.12			**< 0.001**
pN0	31 (54.4)		22 (61.1)	9 (42.9)		19 (90.5)	12 (33.3)	
pN1	15 (26.3)		10 (27.8)	5 (23.8)		2 (9.5)	13 (36.1)	
pN2-pN3	11 (19.3)		4 (11.1)	7 (33.3)		0 (0)	11 (30.6)	
TNM stage					0.13			**< 0.001**
0 - I	13 (22.8)		11 (30.6)	2 (9.5)		9 (42.9)	4 (11.1)	
II	24 (42.1)		15 (41.7)	9 (42.9)		10 (47.6)	14 (38.9)	
III	20 (35.1)		10 (27.8)	10 (47.6)		2 (9.5)	18 (50)	
Angiolymphatic invasion					**0.07**			**0.04**
No	43 (75.4)		30 (83.3)	13 (61.9)		19 (90.5)	24 (66.7)	
Yes	14 (24.6)		6 (16.7)	8 (38.1)		2 (9.5)	12 (33.3)	
P53 IHC*					0.75			0.13
Median (IQR)	0.3 (0–0.7)		0.3 (0–0.7)	0.4 (0–0.7)		0.2 (0–0.4)	0.4 (0,0.7)	
cfDNA information								
Pre-surgical cfDNA concentration					0.99			0.80
Median (IQR) (ng/ml)	24 (15.5–48.8)		25 (14.7–51.2)	21.2 (17.8–29.1)		24.8 (12.9–54.1)	23.5 (17.2–42.2)	
ctDNA mean MAF					**0.04**			0.12
Median (IQR) (%)	0.3 (0.2–1.0)		0.3 (0.1–0.6)	0.7 (0.3–1)		0.3 (0.1–0.6)	0.4 (0.2–1)	
Number of ctDNA variants					**< 0.001**			0.23
Median (IQR)	4 (3–5)		3 (2–4)	5 (4–6)		4 (2–4)	4 (3–6)	
ctDNA TP53 mutation					**0.01**			0.77
No	23 (40.3)		19 (52.8)	4 (19)		9 (42.9)	14 (38.9)	
Yes	34 (59.6)		17 (47.2)	17 (81)		12 (57.1)	22 (61.1)	
ctDNA PIK3CA mutation					0.71			0.34
No	39 (68.4)		24 (66.7)	15 (71.4)		16 (76.2)	23 (63.9)	
Yes	18 (31.6)		12 (33.3)	6 (28.6)		5 (23.8)	13 (36.1)	

cfDNA, cell-free DNA; ctDNA, circulating tumor DNA; *SD, Standard Deviation; IQR, Inter Quartile Range; MAF, mutant allele frequency; NA, Not Available; IHC, Immunohistochemical. ^#^One case with unknown tumor differentiation. The variables with a bold value (p <0.1) were candidate factors used in the Cox univariate analysis.

The median follow-up time was 40.87 months (interquartile range [IQR]: 23.10–61.93). Overall, 64.91% (37/57) of the included participants had a recurrence; in detail, 36.84% (21/57) had a recurrence within 2 years, and 63.16% (36/57) had a recurrence within 5 years. In addition, median DFS was 32.17 months (IQR: 9.77–61.87), and 61.40% (35/57) died during follow-up; among these deaths, 26.32% (15/57) occurred within 2 years, and 59.65% (34/57) occurred within 5 years.

Ivor–Lewis esophagectomy was performed in 75.5% of the patients. The average maximal longitudinal diameter of the tumor was 3.62 ± 1.56 cm, and the median tumor volume was 9 cm^3^. In 70.18% of cases, the lesions were located in the middle thoracic region of the esophagus. Further, 64.9% of the patients had moderately differentiated tumors (lymph node metastasis, 45.6%; angiolymphatic invasion, 24.6%). According to the AJCC/Union for International Cancer Control staging criteria (8th edition), three cases (5.3%) had carcinoma in situ, and 10 (17.5%), 24 (42.1%), and 20 (35.1%) cases had stage I, stage II, and stage III tumors, respectively. The median preoperative plasma cfDNA concentration, average mutant allele frequency (MAF) of ctDNA variants, and number of ctDNA variants were 24 ng/mL, 0.3%, and 4, respectively (**Table 1**, **Supplementary Table S1**).

There were no statistical differences between the recurrence and non-recurrence groups in average age, sex, body mass index, smoking history, drinking history, tumor volume, tumor differentiation grade, immunohistochemical p53 expression, preoperative cfDNA concentration, or PIK3CA mutation status. However, during the 2-year follow-up period, significant differences were observed between the recurrence and non-recurrence groups in the maximum tumor diameter (*p* = 0.01), mean MAF of ctDNA variants (*p* = 0.04), number of ctDNA variants (*p <*0.001), and ctDNA TP53 mutation status (*p* = 0.01). In addition, during the 5-year follow-up period, significant differences were observed between the recurrence and non-recurrence groups in tumor location (*p* = 0.02), surgical procedure (*p* = 0.03), maximum tumor diameter (*p* = 0.03), depth of invasion (*p* = 0.004), lymph node stage (LNS) (*p <*0.001), TNM stage (*p* < 0.001), and angiolymphatic invasion (*p* = 0.04) (**Table 1**).

### 3.2 Mutational landscape of ESCC patients

**Figure 2:**
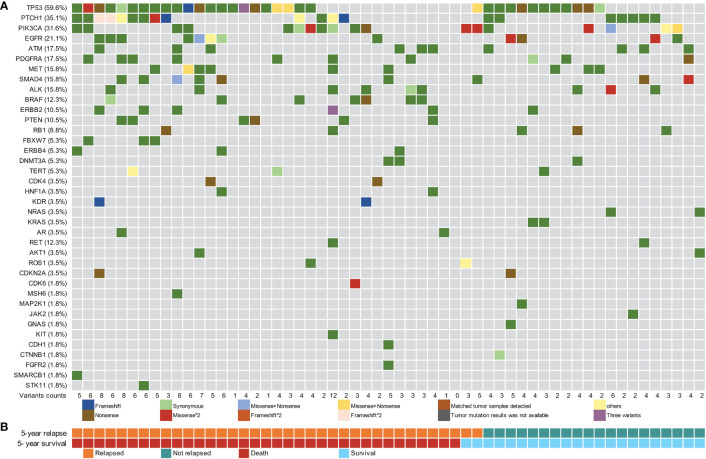
Pre-surgical circulating tumor DNA mutation signature of 57 patients with esophageal squamous cell carcinoma. **(A)** Pre-surgical circulating tumor DNA (ctDNA) mutational landscape of 57 patients. **(B)** The 5-year recurrence and survival status of patients.

Using a 61-gene panel, we analyzed 53 tDNA samples from patients with ESCC and 57 preoperative plasma cfDNA and leukocyte gDNA samples. The average sequencing depths of tumor FFPE and pre-surgical cfDNA samples were 530× and 9275×, respectively; 73.6% (39/53) of the ESCC tissue specimens showed at least one mutation. In total, 73 tumor-specific somatic mutations were detected in 16 genes from 39 patients, including genes with recurrent somatic alterations, such as *TP53* (62.3%), *PIK3CA* (17.0%), *EGFR* (7.5%), and *PTCH1* (5.7%) (**Figure S1**). Among the 57 preoperative cfDNA samples, only one had no ctDNA mutation, and 227 variants were detected in 38 genes. The cfDNA samples had more variants than the tDNA samples. The genes with recurrent somatic alterations in ctDNA were *TP53* (59.6%), *PTCH1* (35.1%), *PIK3CA* (31.6%), and *EGFR* (21.1%) (**Figure 2**). Among the 73 variants detected in the FFPE samples, 49 mutations were also detected in the corresponding cfDNA samples, and the overall sensitivity of cfDNA detection was 67.1% (49/73). Among the 39 patients with detectable tissue variants, at least one mutation was also detected in 29 corresponding cfDNA samples, yielding an overall sensitivity of 74.3% (29/39) (**Figure S1**).

The mean MAF of ctDNA in recurrence cases within two years was higher than that in censored cases (**Figure S2**). The median MAF of the 227 ctDNA variants detected was 0.20% (IQR: 0.11%–0.48%), and 85.0% (193/227) of the variants had a MAF of <1% (**Figure S3A**). The MAF of ctDNA variants was significantly lower than that of tDNA variants, with a difference in one to two orders of magnitude (**Figures S3A, S3B**). The median MAF values of ctDNA variants in which the matched tDNA samples were detected (tDNA+) and those of ctDNA variants in which the matched tDNA samples were not detected (tDNA-) were 0.76% (IQR: 0.32%–2.05%) and 0.17% (IQR: 0.10%–0.29%), respectively; the difference in MAF values between the two groups was statistically significant (*p <*0.001, **Figure S3C**).

### 3.3 ctDNA features and survival

**Figure 3:**
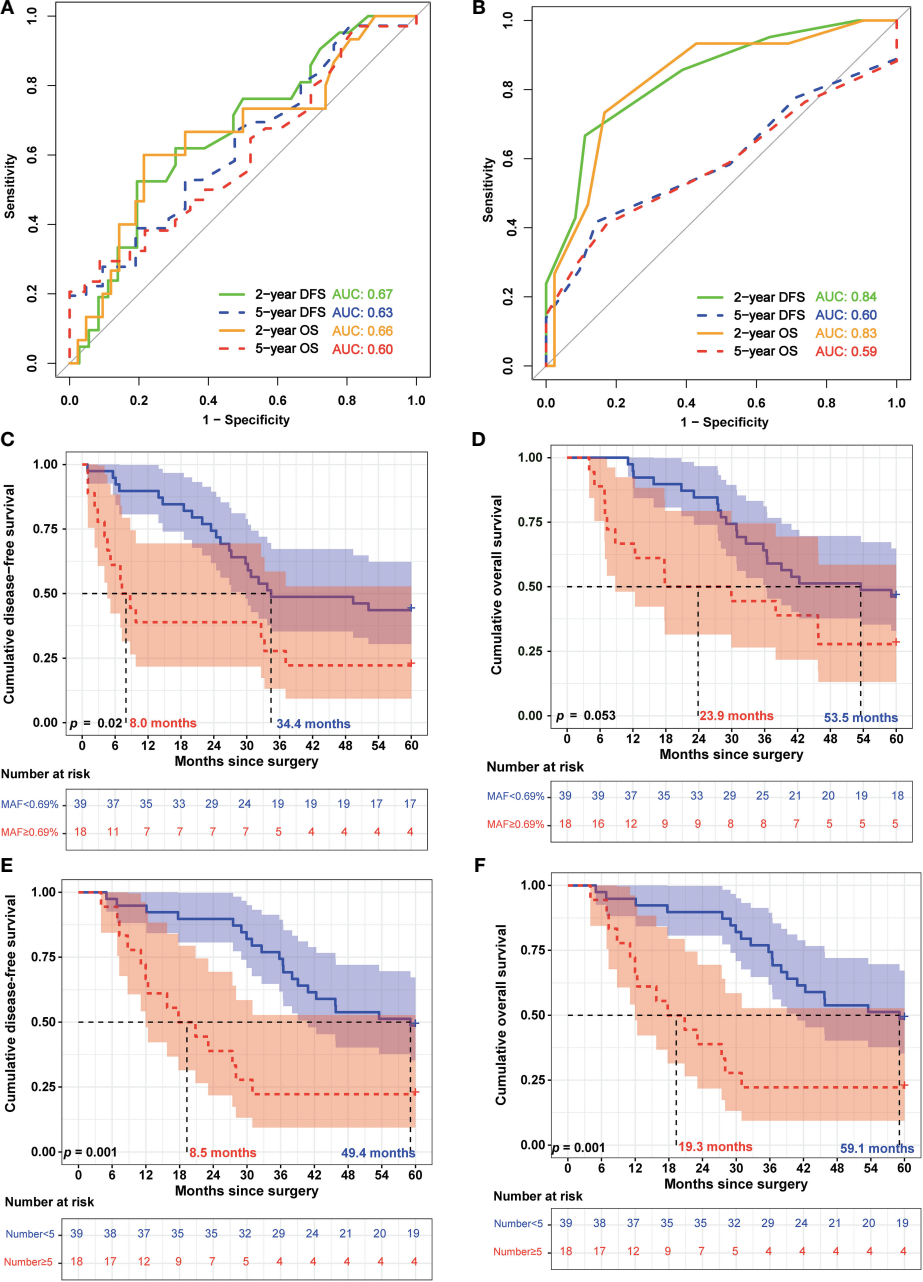
Circulating tumor DNA features were associated with the prognosis of patients with esophageal squamous cell carcinoma. **(A)** Receiving operating characteristic (ROC) curves were used to assess the efficiency of the mean circulating tumor DNA (ctDNA) mutant allele frequency (MAF) for predicting 2-year and 5-year disease-free survival (DFS) and overall survival (OS). **(B)** ROC curves of the number of ctDNA variants for predicting 2- and 5-year DFS and OS. **(C)** Kaplan–Meier analysis of DFS of patients with esophageal squamous cell carcinoma (ESCC) was stratified by mean ctDNA MAF. **(D)** Kaplan–Meier analysis of OS of patients with ESCC was stratified by mean ctDNA MAF. **(E)** Kaplan–Meier analysis of DFS of patients with ESCC was stratified by the number of ctDNA variants. **(F)** Kaplan–Meier analysis of OS of patients with ESCC was stratified by the number of ctDNA variants.

Additionally, we analyzed the relationship between ctDNA features and survival. The receiver operating characteristic (ROC) curve analysis showed that the mean MAF of ctDNA and the number of ctDNA variants could potentially predict recurrence and death in patients with EC (**Figures 3A, B**). In particular, the AUC reached 0.84 and 0.83 (**Figure 3B**) when the number of ctDNA variants was used to predict the 2-year DFS and OS, respectively. Subsequently, the patients were divided into low-risk and high-risk groups according to the optimal thresholds obtained using the Youden index from the ROC curve. The best cutoff value for the mean MAF of ctDNA was 0.69%, with which 68.4% (39/57) and 31.6% (18/57) of patients were categorized into the low-risk and high-risk groups, respectively (**Figures 3C, D**). The difference in the median DFS between the low- and high-risk groups was statistically significant (8.0 vs. 34.4 months; *p* = 0.02) (**Figure 3C**). However, the difference in the median OS between the low- and high-risk groups was not statistically significant (23.9 vs. 53.5 months; *p* = 0.053) (**Figure 3D**). The best cutoff value for the number of ctDNA variants was 5, with which 68.4% (39/57) and 31.6% (18/57) of patients were categorized into the low-risk and high-risk groups, respectively (**Figures 3E, F**). Notably, the differences in both median DFS and OS between the low- and high-risk groups were statistically significant (DFS: 8.5 vs. 49.4 months, *p* = 0.001; OS: 19.3 vs. 59.1 months, *p* = 0.001; **Figures 3E, F**).

### 3.4 Predictors of ESCC prognosis

Cox regression analysis was used to analyze the factors related to DFS and OS in EC. The variables with a *p <*0.1 (**Table 1**) were included as candidate factors in the Cox univariate analysis. The univariate analysis showed that drinking history, tumor location, depth of submucosal invasion, LNS, angiolymphatic invasion status, number of preoperative ctDNA variants, and mean MAF of ctDNA were prognostic factors associated with DFS. Further, the drinking history, tumor location, surgical procedure, depth of submucosal invasion, LNS, angiolymphatic invasion status, and number of preoperative ctDNA variants were prognostic factors associated with OS. We incorporated the variables with *p <*0.05 into the Cox multivariate regression analysis. The multivariate analysis showed that the LNS (*p <*0.05), mean MAF of ctDNA (HR: 2.29; 95% CI: 1.0–5.2; *p* = 0.048), and number of ctDNA variants (*p <*0.01) were independent prognostic factors associated with DFS. Further, the drinking history (HR: 2.77; 95% CI: 1.04–7.35; *p* = 0.04), LNS (*p <*0.05), angiolymphatic invasion status (HR: 2.62; 95% CI: 1.01–6.77), and number of preoperative ctDNA variants (*p <*0.05) were independent prognostic factors associated with OS (**Table 2**).

**Table 2:** Cox regression analysis of predictors for DFS and OS of ESCC patients.

Variables	Univariate analysis				Multivariate analysis			
	Disease-free survival		Overall survival		Disease-free survival		Overall survival	
	*P*	HR (95% CI)	*P*	HR (95% CI)	*P*	HR (95% CI)	*P*	HR (95% CI)
Drinking history (Yes vs. No)	**0.04**	2 (1.03–3.86)	**0.01**	2.38 (1.19–4.72)	***-***	–	***0.04***	2.77 (1.04–7.35)
Tumor location (Middle-upper vs. Lower)	**<0.01**	0.37 (0.18–0.74)	**<0.01**	0.31 (0.15–0.63)	***-***	–	***-***	–
Surgical procedure								
Mckeown vs. Ivor–Lewis	0.07	0.34 (0.1–1.11)	**0.04**	0.22 (0.05–0.91)	***-***	–	***-***	–
Sweet vs. Ivor–Lewis	0.14	2.22 (0.77–6.41)	**0.04**	3.12 (1.05–9.27)	***-***	–	***-***	–
Submucosal invasion								
pT2 vs. pTis-T1	0.25	1.83 (0.65–5.14)	0.17	2.19 (0.7–6.81)	***-***	–	***-***	–
pT3 vs. pTis-T1	**<0.01**	4.27 (1.57–11.60)	**<0.01**	5.03 (1.69–14.95)	***-***	–	***-***	–
Lymph node stage								
pN1 vs. pN0	**<0.01**	3.02 (1.36–6.67)	**0.01**	2.93 (1.28–6.68)	**0.03**	3.83 (1.11–13.15)	**0.017**	5.94 (1.38–25.63)
pN2-pN3 vs. pN0	**<0.001**	6.93 (2.92–16.4)	**<0.001**	5.79 (2.46–13.60)	**<0.01**	9.18 (1.87–45.21)	**0.014**	10.38 (1.62–66.5)
Angiolymphatic invasion (Yes vs. No)	**<0.01**	2.72 (1.35–5.48)	**<0.01**	2.82 (1.37–5.76)	***-***	–	**0.047**	2.62 (1.01–6.77)
ctDNA mean MAF (≥0.69% vs. < 0.69%)	**0.02**	2.23 (1.14–4.37)	0.06	1.96 (0.97–3.92)	**0.048**	2.29 (1–5.2)	***-***	–
ctDNA variants number								
5–6 vs. 0–4	**<0.01**	2.8 (1.3–6.02)	**0.04**	2.33 (1.05–5.15)	**<0.01**	4.3 (1.63–11.33)	**0.018**	3.91 (1.26–12.08)
> 6 vs. 0–4	**<0.001**	9.94 (3.3–29.97)	**<0.001**	8.1 (2.83–23.17)	**<0.001**	17.14 (4.39–66.9)	**<0.001**	70.53 (11.67–426.22)
cfDNA TP53 mutation (Yes vs. No)	0.32	1.41 (0.72–2.75)	0.34	1.40 (0.70–2.81)	***-***	–	***-***	–

cfDNA, cell-free DNA; ctDNA, circulating tumor DNA; DFS, Disease-free Survival; MAF, mutant allele frequency; OS, Overall Survival; ESCC, Esophageal Squamous Cell Carcinoma. The bold values indicate that the difference is statistically significant (p < 0.05).

### 3.5 Prediction model of disease-free survival

**Figure 4:**
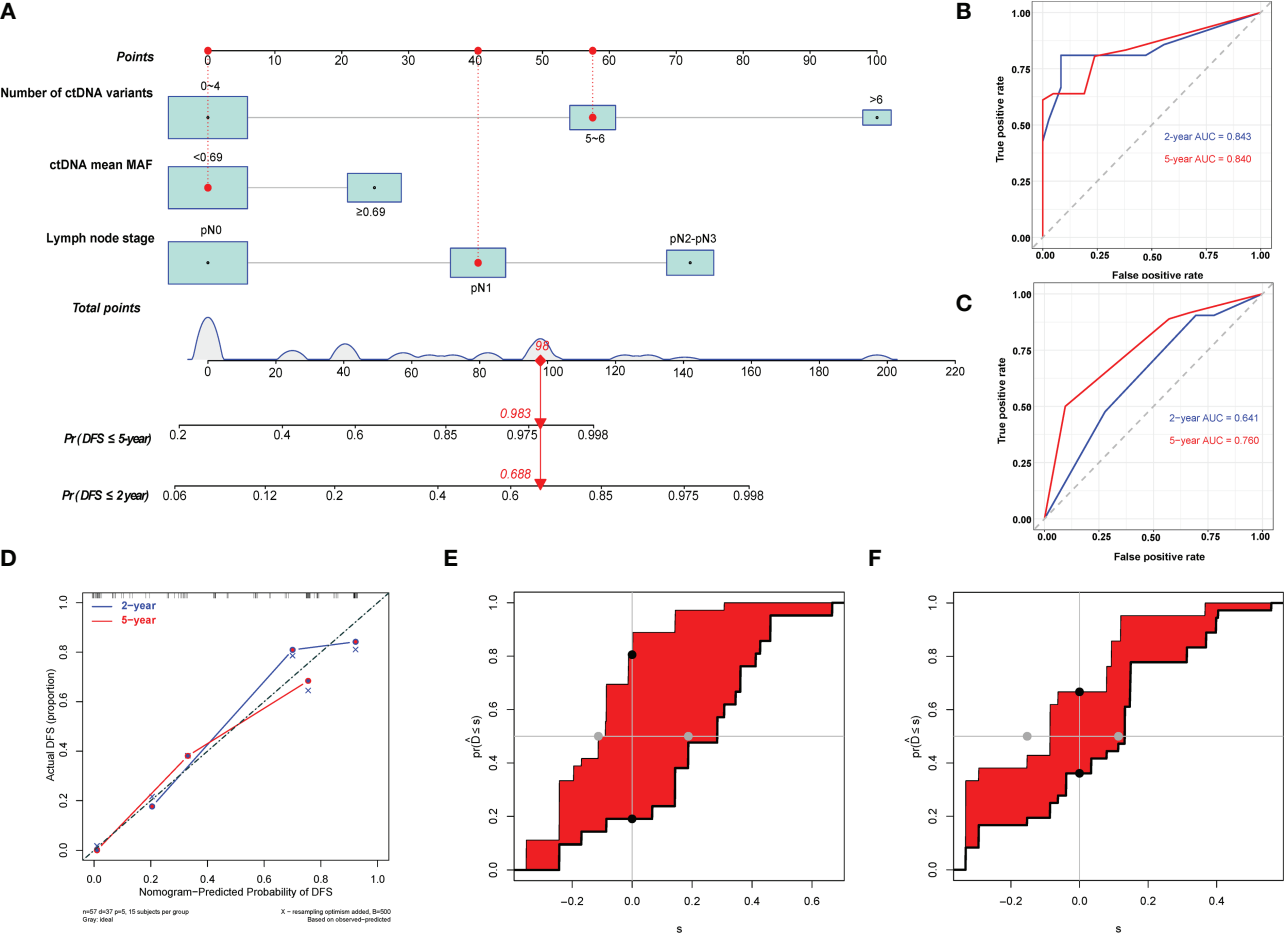
Development and evaluation of nomogram for disease-free survival prediction in patients with esophageal squamous cell carcinoma. **(A)** Nomogram based on preoperative circulating tumor DNA features for disease-free survival (DFS) in patients with resectable esophageal squamous cell carcinoma. The prediction result of patient no. 28 is listed as an example. Each subgroup within the variables was assigned a score. To determine the score of each independent prognostic factor, a vertical line was drawn from each factor axis to the “Points” scale; the position of the red dot on the “Points” axis indicates the score of this factor (pN1 LNS, score = 40; mean ctDNA MAF = 0.31%, <0.69%, score = 0; and six variants of ctDNA, score = 58). The predicted probability of DFS can be found vertically below the location of the Total Points. The total scores for all three predictors were summed to get the “Total points” value (40 + 0+58 = 98, which is indicated as a red diamond on the “Total points” scale). The red arrow on the “Pr” axis indicates the recurrence probability in the corresponding years (the recurrence probability within two years was 0.688, and the recurrence probability within five years was 0.983). **(B)** Receiving operating characteristic (ROC) curves of the nomogram for predicting 2-year and 5-year DFS. **(C)** ROC curves of the TNM staging system for predicting 2-year and 5-year DFS. **(D)** Calibration plot of the nomogram for predicting DFS. **(E)** Integrated discrimination improvement (IDI) between the developed nomogram and the TNM staging system for predicting 2-year DFS. **(F)** IDI between the developed nomogram and the TNM staging system for predicting 5-year DFS.

We used the three identified independent factors associated with EC DFS as candidate variables for the nomogram (**Figure 4A**). The prognostic scores for the DFS of each subgroup within the variables are shown in **Supplementary Table S3**. As an example, patient no. 28 had a pN1 LNS (score = 40), a mean ctDNA MAF of 0.31% (<0.69%, score = 0), and six ctDNA variants (score = 58). The patient’s total score was 98, and the model predicted that the probabilities of recurrence within 2 and 5 years were 0.688 and 0.983, respectively (**Figure 4A**). Consistent with the nomogram model-predicted results, clinical imaging confirmed recurrence at 20.03 months after surgery in this patient.

The C-indices of the model for predicting 2- and 5-year DFS were 0.82 (95% CI: 0.74–0.91) and 0.78 (95% CI: 0.70–0.86), respectively (**Table 3**), while those of the TNM staging system for predicting 2- and 5-year DFS were 0.64 (95% CI: 0.53–0.75) and 0.65 (95% CI: 0.56–0.74), respectively. In addition, the AUCs of the nomogram for predicting 2- and 5-year DFS were 0.84 (95% CI: 0.72–0.97) and 0.84 (95% CI: 0.74–0.94), respectively (**Figure 4B**, **Table 3**), whereas those of the TNM staging system for predicting 2- and 5-year DFS were 0.64 (95% CI: 0.50–0.78) and 0.76 (95% CI: 0.64–0.88), respectively (**Figure 4C**, **Table 3**). After setting the number of bootstrap resamplings to 500, the calibration curve demonstrated a favorable consistency between the actual 2- and 5-year DFS and nomogram-predicted results (**Figure 4D**). Furthermore, the Hosmer–Lemeshow test showed that the predicted recurrences within 2 (*p* = 0.12) and 5 years (*p* = 0.40) both had *p >*0.05, indicating consistency between the observed and predicted DFS. Compared with the IDI of the TNM staging system, that of the established nomogram model was 0.33 (95% CI: 0.18–0.49; *p <*0.001) (**Figure 4E**, **Table 3**) for 2-year DFS prediction and 0.18 (95% CI: 0.02–0.29; *p* = 0.04) (**Figure 4F**, **Table 3**) for 5-year DFS prediction.

**Table 3:** Comparison of performance between nomogram model and the TNM staging system in predicting DFS of ESCC patients.

Performance	Two-year DFS* (value, 95% CI)		Five-year DFS (value, 95% CI)	
	Nomogram model	TNM staging system	Nomogram model	TNM staging system
C-index	0.82 (0.74–0.91)	0.64 (0.53–0.75)	0.78 (0.70–0.86)	0.65 (0.56–0.74)
AUC*	0.84 (0.72–0.97)	0.64 (0.50–0.78)	0.84 (0.74–0.94)	0.76 (0.64–0.88)
Sensitivity (%)	80.95 (58.09–94.55)	90.48 (69.62–98.83)	61.11 (43.46–76.86)	50 (32.92–67.08)
Specificity (%)	91.67 (77.53–98.25)	30.56 (16.35–48.11)	100 (83.89–100.00)	90.48 (69.62–98.83)
PPV* (%)	85 (65.28–94.47)	43.18 (37.01–49.57)	100	90 (69.83–97.22)
NPV* (%)	89.19 (77.26–95.25)	84.62 (57.38–95.74)	60 (49.90–69.32)	51.35 (42.53–60.08)
Accuracy (%)	87.72 (76.32–94.92)	52.63 (38.97–66.02)	75.44 (62.24–85.87)	64.91 (51.13–77.09)
IDI*	0.33 (95% CI: 0.18–0.49, p < 0.001)		0.18 (95% CI: 0.02–0.29, p = 0.04)	

*DFS, Disease-free Survival; ESCC, Esophageal Squamous Cell Carcinoma; 95% CI, 95% Confidence Interval; AUC, Area Under Curve; PPV, Positive Predictive Value; NPV, Negative Predictive Value; IDI, Integrated Discrimination Improvement.

**Figure 5:**
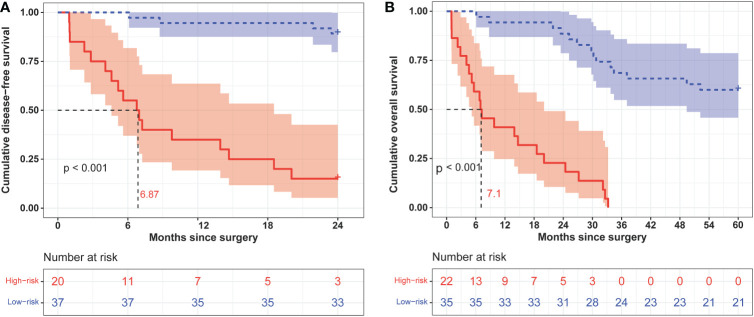
Kaplan–Meier curves of 2-year **(A)** and 5-year **(B)** disease-free survival in different risk groups classified according to nomogram scores.

We calculated the comprehensive scores for the 2- and 5-year recurrence probabilities of each case according to the nomogram (**Supplementary Table S4**). Based on the Youden index from the ROC analysis, a total score of 77 was used as the threshold for the 2-year recurrence risk stratification. A score of ≥77 indicated a high risk of recurrence within 2 years, while a score of <77 indicated a low risk of recurrence within 2 years. The median DFS of the high and low-risk groups was 6.87 months and undefined, respectively (**Figure 5A**), and the sensitivity, specificity, and accuracy of the predictions were 80.95%, 91.67%, and 87.72%, respectively. In contrast, the sensitivity, specificity, and accuracy of the TNM staging system in predicting the 2-year DFS were 90.48%, 30.56%, and 52.63%, respectively (**Table 3**). In addition, a total score of 68.5 was used as the threshold for the 5-year recurrence risk stratification. A score of ≥68.5 indicated a high risk of recurrence within 5 years, while a score of <68.5 indicated a low risk of recurrence within 5 years. The median DFS of high- and low-risk groups was 7.1 months and undefined (**Figure 5B**), respectively, and the sensitivity, specificity, and accuracy of predictions were 61.11%, 100%, and 75.44%, respectively (**Table 3**). In contrast, the sensitivity, specificity, and accuracy of the TNM staging system in predicting the 5-year DFS were 50%, 90.48%, and 64.91%, respectively (**Table 3**).

### 3.6 Prediction model of overall survival

**Figure 6:**
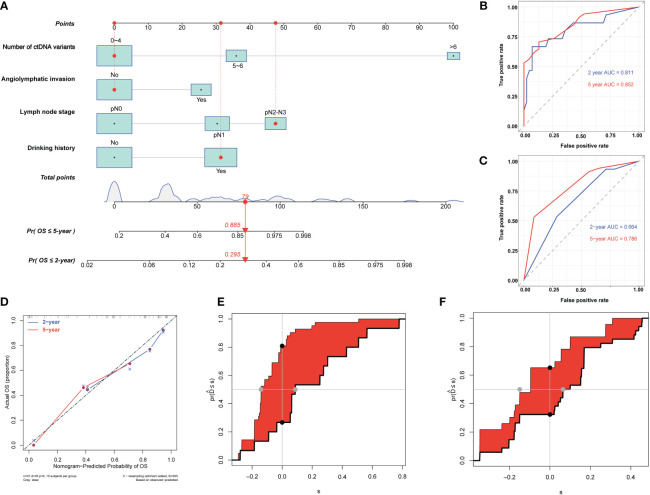
Development and evaluation of nomogram for overall survival prediction in patients with esophageal squamous cell carcinoma (ESCC). **(A)** Nomogram based on preoperative circulating tumor DNA features for overall survival (OS) in patients with resectable ESCC. The prediction result of patient no. 15 is listed as an example. Each subgroup within the variables was assigned a score. To determine the score of each independent prognostic factor, a vertical line was drawn from each factor axis to the “Points” scale; the position of the red dot on the “Points” axis indicates the score of this factor (with a drinking history, score = 31; no angiolymphatic invasion, score = 0; pN2 LNS, score =48; and three variants of ctDNA, score = 0). The predicted probability of OS can be found vertically below the location of the Total Points. The total scores for all four factors are summed to get the “Total points” value (31 + 0+48+0 = 79, which is indicated as a red diamond on the “Total points” scale). The red arrow on the “Pr” axis indicates the recurrence probability in the corresponding years (the recurrence probability within two years was 0.295, and the recurrence probability within five years was 0.885). **(B)** Receiving operating characteristic (ROC) curves of the nomogram for predicting 2-year and 5-year OS. **(C)** ROC curves of the TNM staging system for predicting 2-year and 5-year OS. **(D)** Agreement between the observed and predicted proportions of OS after surgery for ESCC patients. **(E)** Integrated discrimination improvement (IDI) between the developed nomogram and TNM staging system for predicting 2-year OS. **(F)** IDI between the developed nomogram and TNM staging system for predicting 5-year OS.

We used the identified four independent risk factors associated with ESCC OS as candidate variables for the nomogram (**Figure 6A**). The prognostic scores for the OS of each subgroup within the variables are shown in **Supplementary Table S3**. As an example, patient no. 15 had a drinking history (score = 31), no angiolymphatic invasion (score = 0), a pN2 LNS (score = 48), and three ctDNA variants (score = 0). The patient’s total score was 79, and the model predicted that the probabilities of death within 2 and 5 years were 0.295 and 0.885, respectively (**Figure 6A**). Consistent with the nomogram model-predicted results, the patient died 32.17 months after surgery.

The C-indices of the nomogram for predicting 2- and 5-year OS were 0.80 (95% CI: 0.68–0.92) and 0.77 (95% CI: 0.68–0.85), respectively (**Table 4**), while those of the TNM staging system for predicting 2- and 5-year OS were 0.66 (95% CI: 0.54–0.78) and 0.66 (95% CI: 0.57–0.74), respectively (**Table 4**). In addition, the AUCs of nomogram for predicting 2- and 5-year OS were 0.81 (95% CI: 0.67–0.95) and 0.85 (95% CI: 0.76–0.95), respectively (**Figure 6B**, **Table 4**), whereas those of the TNM staging system for predicting 2- and 5-year OS were 0.66 (95% CI: 0.52–0.81) and 0.79 (95% CI: 0.68–0.9), respectively (**Figure 6C**, **Table 4**). After setting the number of bootstrap resamplings to 500, the calibration curves demonstrated a favorable consistency between the actual 2-year and 5-year OS and nomogram-predicted results (**Figure 6D**). Moreover, the Hosmer–Lemeshow test showed that the predicted death within 2 (*p* = 0.73) and 5 years (*p* = 0.65) both had *p >*0.05, indicating a favorable consistency between the observed and predicted OS. Compared with IDI of the TNM staging system, that of the established nomogram was 0.28 (95% CI: 0.11–0.46; *p <*0.001) for the 2-year OS prediction (**Figure 6E**, **Table 4**) and 0.15 (95% CI: 0–0.36; *p* = 0.04) for the 5-year OS prediction (**Figure 6F**, **Table 4**).

**Table 4:** Comparison of performance between the nomogram model and the TNM staging system in predicting OS of ESCC patients.

Performance	Two-year OS (value, 95% CI)		Five-year OS (value, 95% CI)	
	Nomogram model	TNM staging system	Nomogram model	TNM staging system
C-index	0.8 (0.68–0.92)	0.66 (0.54–0.78)	0.77 (0.68–0.85)	0.66 (0.57–0.74)
AUC	0.81 (0.67–0.95)	0.66 (0.52–0.81)	0.85 (0.76–0.95)	0.79 (0.68–0.9)
Sensitivity	66.67 (38.38–88.18)	53.33 (26.59–78.73)	70.59 (52.52–84.90)	52.94 (35.13–70.22)
Specificity	92.86 (80.52–98.50)	71.43 (55.42–84.28)	86.96 (66.41–97.22)	91.3 (71.96–98.93)
PPV	76.92 (51.41–91.31)	40 (25.38–56.65)	88.89 (73.15–95.92)	90 (69.75–97.23)
NPV	88.64 (79.14–94.13)	81.08 (70.71–88.38)	66.67 (53.72–77.51)	56.76 (47.35–65.70)
Accuracy	85.96 (74.21–93.74)	66.67 (52.94–78.60)	77.19 (64.16–87.26)	68.42 (54.76–80.09)
IDI	0.28 (95% CI: 0.11–0.46, p < 0.001)		0.15 (95% CI: 0–0.36, p = 0.04)	

OS, Overall Survival; ESCC, Esophageal Squamous Cell Carcinoma; 95% CI, 95% Confidence Interval; AUC, Area Under Curve; PPV, Positive Predictive Value; NPV, Negative Predictive Value; IDI, Integrated Discrimination Improvement.

**Figure 7:**
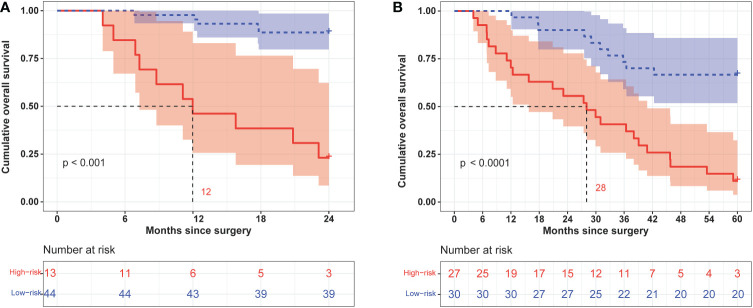
Kaplan–Meier curves of 2-year **(A)** and 5-year **(B)** overall survival in different risk groups classified according to the nomogram score.

We calculated the comprehensive scores of the 2- and 5-year death probabilities of each case according to the nomogram (**Supplementary Table S4**). Based on the Youden index from the ROC analysis, a total score of 94.5 was used as the threshold for the 2-year survival risk stratification. A score of ≥94.5 indicated a high risk of death within 2 years, while a score of <94.5 indicated a low risk of death within 2 years. The median OS of the high- and low-risk groups was 12.0 months and undefined (**Figure 7A**), respectively, and the sensitivity, specificity, and accuracy of the predictions were 66.67%, 92.86%, and 85.96%, respectively. In contrast, the sensitivity, specificity, and accuracy of the TNM staging system in predicting the 2-year OS were 53.33%, 71.43%, and 66.67%, respectively. In addition, a comprehensive score of 42 was used as the threshold for the 5-year survival risk stratification. A score of ≥42 indicated a high risk of death within 5 years, while a score of <42 indicated a low risk of death within 5 years. The median OS of the high- and low-risk groups was 28.0 months and undefined, respectively (**Figure 7B**), and the sensitivity, specificity, and accuracy of the predictions were 70.59%, 86.96%, and 77.19%, respectively (**Table 4**). In contrast, the sensitivity, specificity, and accuracy of the TNM staging system in predicting the 5-year OS were 52.94%, 91.3%, and 68.42%, respectively (**Table 4**).

## 4 Discussion

Patients with EC have a poor prognosis. Therefore, early prediction of prognosis in patients with EC is crucial for the selection of treatment strategies. In this study, we analyzed ctDNA features and pathological markers through Cox univariate and multivariate analyses and successfully established a predictive nomogram to forecast DFS and OS in patients with resectable ESCC. Compared with the traditional TNM staging system, our nomogram model showed better performance in terms of predictive accuracy and discriminative ability in the prognosis of patients with ESCC. The comprehensive scores from the nomogram could efficiently stratify patients into high- and low-risk subgroups based on DFS and OS.

In recent years, various nomogram models have been developed for EC (**Table S5**). Nomograms based on traditional demographic characteristics (such as age, sex, and race) and clinicopathological features (such as TNM stage, tumor location, tumor size, tumor grade, depth of invasion, and number of metastases) only show a moderate performance (16, 19, 20). Some studies have incorporated the nutritional information and inflammatory profile of patients into the nomogram and successfully demonstrated that the developed nomogram has a better performance than the TNM staging system (6, 21, 22). In addition, other studies have incorporated genomic data, such as DNA methylation (23), long non-coding RNA (24), and autophagy-related gene data (25), into the nomogram and successfully shown that the developed nomogram has a better discrimination ability than the TNM staging system. However, no studies have incorporated ctDNA mutation information into nomogram models. To the best of our knowledge, our study is the first to incorporate ctDNA mutation features into a nomogram to establish a predictive model of postoperative survival in EC.

ctDNA has been widely used in clinical research to predict tumor size, tumor stage, minimal residual lesions, tumor recurrence and metastasis, survival, and follow-up treatment (26–28). Studies have demonstrated that the mean MAF of ctDNA at baseline is associated with tumor size and breast cancer stage (29), and MAF of ctDNA positively correlates with tumor burden in colorectal cancer (30); in addition, high ctDNA molecule numbers, a high number of mutations, and specific variants are associated with poor outcomes in breast cancer (31); moreover, high number of ctDNA baseline variants before chemotherapy predicted unfavorable outcome in patients with metastatic gastroesophageal cancer (32). However, few studies have investigated ctDNA features in EC than in colorectal and breast cancers; thus, more studies are needed to reveal the characteristics of ctDNA and their association with prognosis in EC. In this study, we used Cox univariate and multivariate analyses to evaluate the association between the mutational signatures of ctDNA and prognosis in EC and found that ctDNA features could reflect tumor load, the average MAF of ctDNA, and the number of ctDNA variants, which are independent risk factors for ESCC. Moreover, the C-indices for 5-year DFS and OS prediction by TNM staging were 0.65 (95% CI: 0.56–0.74) and 0.66 (95% CI: 0.57–0.74), respectively, consistent with the values reported in other studies (20, 22, 33, 34) (**Supplementary Table S5**).

Our study has the following strengths (1): To a certain extent, we revealed the mutational characteristics of ctDNA in EC, which may be beneficial to the research on ctDNA in EC. (2) In our study, we combined not only common pathological data but also ctDNA features to establish a prediction model for postoperative survival in EC. (3) Our model showed good performance in predicting both DFS and OS, with 5-year indices of 0.78 and 0.77, respectively. Compared with the traditional TNM staging system, our nomogram showed better predictive ability and accuracy in the prognosis of patients with ESCC. The IDI for 2-year DFS and OS was 0.33 (95% CI: 0.18–0.49; *p <*0.001) and 0.28 (95% CI: 0.11–0.46; *p <*0.001), respectively. Our nomogram can be used for individual survival prediction and help clinicians choose appropriate treatment strategies.

However, our study has the following limitations: (1) The sample size was small. Although we enrolled 75 patients in this study, only 57 were included in the data analysis. (2) ctDNA detection is still in the clinical research stage. Sequencing depth and panel selection vary among various clinical trials, and the cost of NGS is higher than that of traditional pathological methods. (3) Since we were unable to obtain more ctDNA mutation information, the new nomogram was only self-verified. As such, the lack of multicenter external verification may affect the applicability of the model. However, the development and standardization of DNA detection technology can potentially address this problem. To further improve the accuracy of the model, more external data must be included in the follow-up. In addition, to further verify the accuracy of the model, more predictive factors must be identified and evaluated (5). Neoadjuvant therapy combined with surgery remains the standard treatment for patients with locally advanced EC. However, tumor treatment, such as radiotherapy and chemotherapy, can easily induce clonal hematopoiesis-related mutations and increase the false-positive probability of ctDNA detection (35). As such, patients who received neoadjuvant therapy were excluded from this study to eliminate the interference of neoadjuvant therapy on ctDNA detection. Supplementing the relevant data of patients receiving neoadjuvant therapy and immunotherapy is necessary for further analysis and verification in the follow-up study. Therefore, information on treatment strategies and responses may be needed for future projections.

In conclusion, this study incorporated easily identifiable prognostic factors into a nomogram model to determine EC prognosis, and the model had good discrimination accuracy and predictive performance. The nomogram model can facilitate individualized prediction of ESCC, help identify high-risk individuals, and potentially guide clinical treatment and follow-up of EC patients in the future. Additionally, the present findings may help further understand the function of ctDNA in patients with ESCC and improve clinical practice.

## Data availability statement

The raw sequencing data presented in this study are deposited in the China National GeneBank (CNGB, https://db.cngb.org/cnsa/), accession numbers CNP0003812 and CNP0001778. According to national legislation, specifically the Administrative Regulations of the People’s Republic of China on Human Genetic Resources, no additional raw data are available at this time. Data from this project can be accessed upon application to CNGB. Please email CNGBdb@cngb.org for detailed application guidance. The accession numbers CNP0003812 and CNP0001778 should be included in the application.

## Ethics statement

The studies involving human participants were reviewed and approved by Review Board of Changhai Hospital. The patients/participants provided their written informed consent to participate in this study.

## Author contributions

TL, ML, and WC contributed equaly to this work. TL contributed to the collection of samples and clinical information and follow-up of patients and assisted in the writing of manuscripts. ML was responsible for analyzing and summarizing data, writing the draft of the manuscripts, and assisting in the follow-up of patients. WC was responsible for sample collection, clinical data acquisition, and draft writing. YX participated in data analysis and manuscript writing. QY was in charge of revising the manuscript. XW contributed to experimental design and implementation and paper modification. HJ contributed to experimental design and organization, as well as analysis with constructive discussions. All authors contributed to the article and approved the submission.
