# Supplementary material for: A clinical prognostic model for patients with esophageal squamous cell carcinoma based on circulating tumor DNA mutation features
Source: Front Oncol. 2023 Jan 5;12:1025284. doi: 10.3389/fonc.2022.1025284 (PMC9850098; doi:10.3389/fonc.2022.1025284)
Supplement: Supplementary file 1 [file DataSheet_1.docx]

Supplementary Material

**A Clinical Prognostic Model for Patients with Esophageal Squamous Cell Carcinoma Based on Circulating Tumor DNA Mutation Features**

**Tao Liu, Mengxing Li, Wen Cheng, Qianqian Yao, Yibo Xue, Xiaowei Wang*, Hai Jin***

**^*^Correspondence:**

Hai Jin**;** [Projinhai@163.com](mailto:Projinhai@163.com)

Xiaowei Wang**;** [drwxw@163.com](mailto:drwxw@163.com)


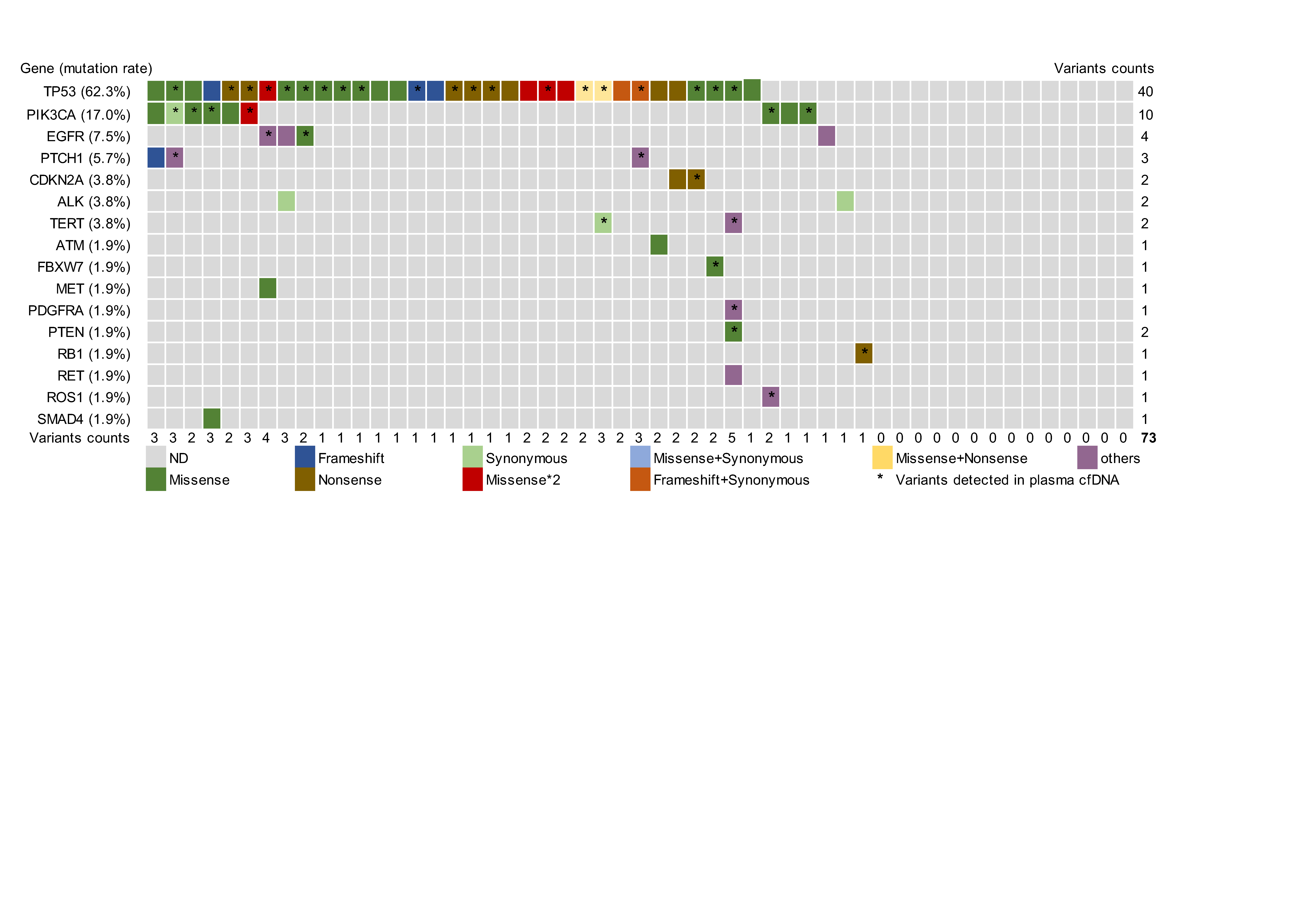


**Figure S1**. Mutational landscape in FFPE tissues of 53 patients with ESCC.

**
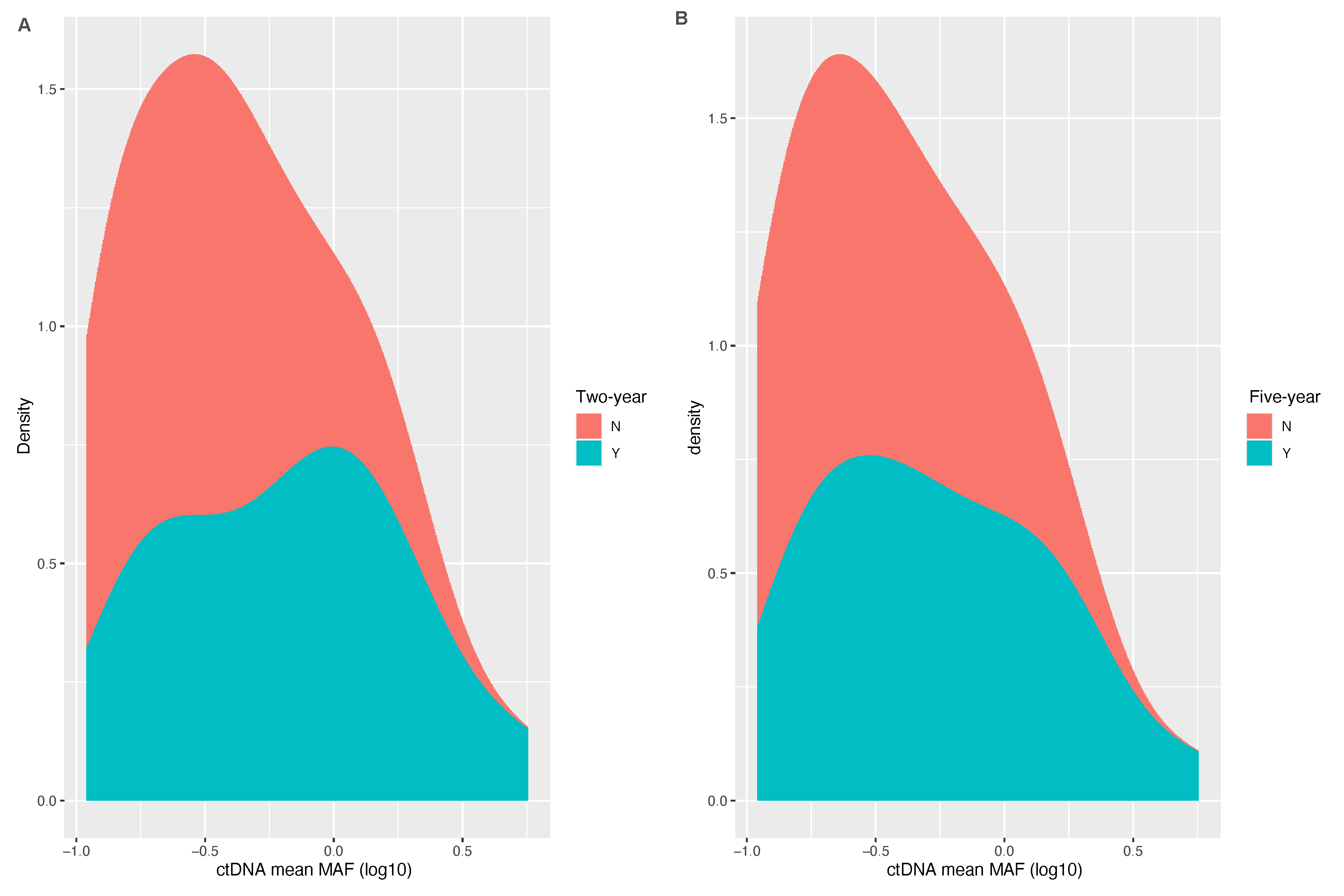
**

**Figure S2**. Density distribution curves of the average mutant allele frequencies of ctDNA variants in 2-year (A) and 5-year (B) recurrence and non-recurrence patients.


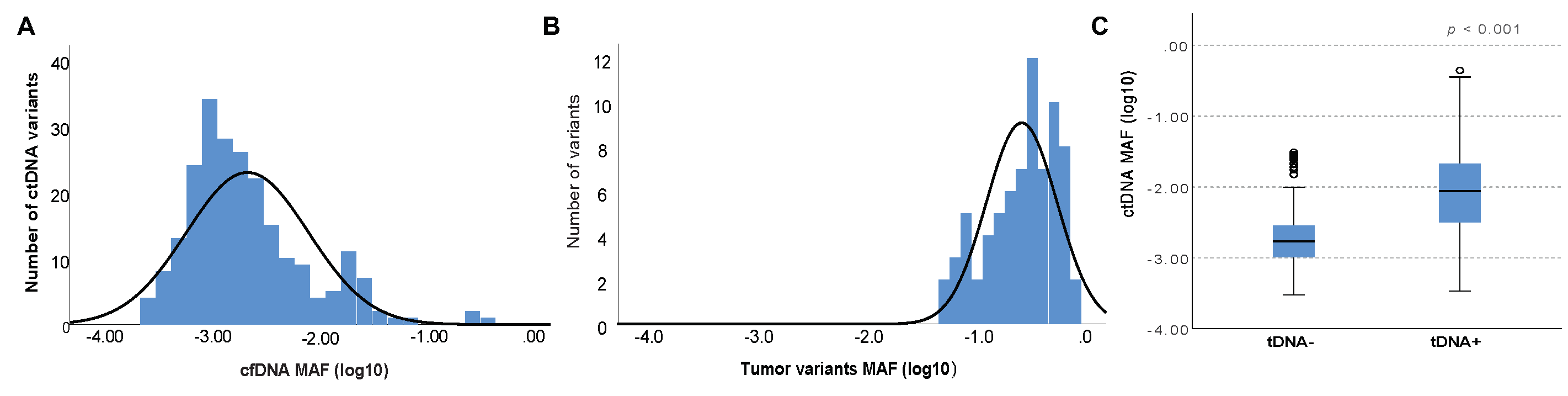


**Figure S3**. Distribution and comparison of mutant allele frequencies in esophageal cancer samples. (A) Distribution histogram of mutant allele frequencies (MAF) of pre-surgical ctDNA variants. (B) Distribution histogram of MAF of tumor variants. Comparison of ctDNA MAF between tumor positive (tDNA+) and tumor negative (tDNA-) groups.

**Supplementary Table S1** Participant inclusion and exclusion criteria.

**Supplementary Table S2** Characteristics of the study cohort.

**Supplementary Table S3** The prognostic scores for DFS and OS of each subgroup within variable.

**Supplementary Table S4** Evaluation of the nomogram model for DFS and OS in two-year and five-year.

**Supplementary Table S5** Comparison of our nomogram model with the reported model.
